# A characteristic biosignature for discrimination of gastric cancer from healthy population by high throughput GC-MS analysis

**DOI:** 10.18632/oncotarget.11754

**Published:** 2016-08-31

**Authors:** Yinan Chen, Jun Zhang, Lei Guo, Lei Liu, Jingran Wen, Lu Xu, Min Yan, Zuofeng Li, Xiaoyan Zhang, Peng Nan, Jinling Jiang, Jun Ji, Jianian Zhang, Wei Cai, Huisheng Zhuang, Yan Wang, Zhenggang Zhu, Yingyan Yu

**Affiliations:** ^1^ Department of Surgery of Ruijin Hospital, and Shanghai Institute of Digestive Surgery, Shanghai Key Laboratory for Gastric Neoplasms, Ruijin Hospital, Shanghai Jiao Tong University School of Medicine, Shanghai, China; ^2^ Tongji University, School of Life Science and Technology, Shanghai, China; ^3^ School of Life Sciences, Fudan University, Shanghai, China; ^4^ School of Environmental Science and Engineering, Shanghai Jiao Tong University, Shanghai, China; ^5^ College of Public Health, Shanghai Jiao Tong University, School of Medicine, Shanghai, China

**Keywords:** gastric cancer, urine, metabolomics, biomarkers, GC-MS

## Abstract

Early diagnosis of gastric cancer is crucial to improve patient′ outcome. A good biomarker will function in early diagnosis for gastric cancer. In order to find practical and cost-effective biomarkers, we used gas chromatography combined mass spectrometer (GC-MS) to profile urinary metabolites on 293 urine samples. Ninety-four samples are taken as training set, others for validating study. Orthogonal partial least squares discriminant analysis (OPLS-DA), significance analysis of microarray (SAM) and Mann-Whitney U test are used for data analysis. The diagnostic value of urinary metabolites was evaluated by ROC curve. As results, Seventeen metabolites are significantly different between patients and healthy controls in training set. Among them, 14 metabolites show diagnostic value better than classic blood biomarkers by quantitative assay on validation set. Ten of them are amino acids and four are organic metabolites. Importantly, proline, p-cresol and 4-hydroxybenzoic acid disclose outcome-prediction value by means of survival analysis. Therefore, the examination of urinary metabolites is a promising noninvasive strategy for gastric cancer screening.

## INTRODUCTION

Gastric cancer ranks the fifth in incidence and the third in mortality among all cancers worldwide [1]. Since there are no specific symptoms at early stage of carcinogenesis, most of patients are diagnosed at advanced stage with lymph node or remote metastasis. Therefore, exploring valuable molecular characterization or biomarkers is desirable for improving current status.

Metabolomics focuses on exploring low-molecular weight metabolites in a biological system [2]. Metabolites are the end products of life activity and always present in body fluids, which are relatively easier to get samples. However, the amount of metabolites is less than that of genes and proteins in cells. For example, Saccharomyces cerevisiae contains over 6000 genes but only 600 metabolites [3]. So it is relatively easy to find out biosignatures from metabolites profile analysis. Metabolites analysis has been applied to the diagnosis of many cancers such as gastric cancer [4, 5], breast cancer [6–9], ovarian cancer [10], pancreatic cancer [11], colorectal cancer [12–15], prostate cancer [16, 17], liver cancer [18, 19], lung cancer [20–23] and bladder cancer [24, 25]. There have been several metabolomics studies on gastric cancer with various platforms such as CE-MS [26], LC-MS [27]and NMR [4, 28]. Up-to-date, most of the studies analyzed small sample sizes and are lack of further validation on a separated cohort. Moreover, none of them could predict the prognosis of gastric cancer. In order to satisfy the clinical demand, more metabolites analysis on large sample size is expected.

GC-MS is widely applied in metabolomics study, which provide a rapid, qualitative and quantitative analyses with the ability to identify small molecule metabolites [29, 30]. GC can separate volatile and thermal stable compounds, then the compounds are eluted and detected by MS. MS is operated by ion formation, separation and detection of ions according to mass-to-charge (m/z) ratio. Qiu and coworkers [29]applied GC-MS analysis on urine detection with ethyl chloroformate (ECF) derivatization method, which was proved stable and repeatable. In the present study, we described a GC-MS-based urinary metabolites profile on a large cohort of gastric cancer. By a precise quantitative assay, we characterized a group of metabolites, which disclosed a diagnostic value for gastric cancer, which are better than that of classic blood tumor biomarkers. Three of metabolites could predict patient′ outcome. The urinary characteristic metabolites may function as promising noninvasive biomarkers/biosignature for gastric cancer screening.

## RESULTS

### Identification of characteristic urinary metabolites on training set

**Figure 1:**
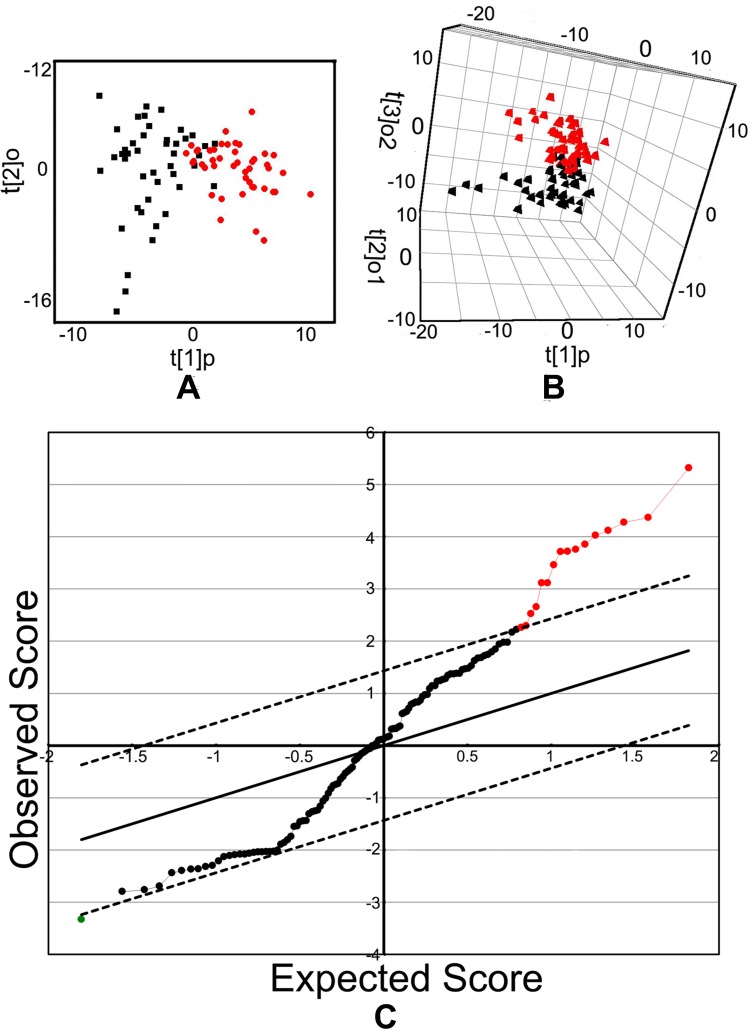
OPLS-DA score plots and SAM analysis plots **A.** OPLS-DA score plots. **B.** OPLS-DA 3D scatter plots. Each symbol represents the metabolomic profile of an individual. The black points represent the controls while the red boxes represent gastric cancer sample. **C.** The 17 variables selected by SAM analysis. The up-regulated variables in cancer group were presented as red spots on the right top, and one down-regulated variable in cancer group was presented as green spot on the left bottom.

A total of 129 metabolites were identified in training set (Supplementary Table 1). The peaks showed high qualities of the raw data with evenly dispersed retention time. By Orthogonal partial least squares discriminant analysis (OPLS-DA), we obtained one predictive component and two orthogonal components (R2Ycum = 0.631;Q2Ycum = 0.509), which could separate the cancer patients from the healthy controls (Figure 1A, 1B). The differential metabolites were identified with the criteria of variable importance in the project values (VIP) > 1. In addition, SAM method and Mann-Whitney U test were also used for metabolites selection. For instance, 17 metabolites were characterized between two groups by SAM method (Figure 1C), which were well overlapped with those identified in OPLS-DA model. At SAM method, 16 metabolites are increased metabolites, and one is decreased metabolite in cancer group (Table 1). By Mann-Whitney U test, the candidate metabolites showed statistic significance (P < 0.01).The metabolites phenylalanine, carbamic acid and 2, 3-octanedione were excluded because of low match percentage with standard mass spectrum. Metabolites p-cresol and benzylmalonic acid were enrolled as their VIP > 1 in OPLS-DA model and were assumed to be associated with environmental pollution. A total of 17 metabolites were determined as candidate molecules for further validation. They were alanine, glycine, isoleucine, valine, proline, serine, threonine, methionine, tyrosine, tryptophan, hippuric acid, ethyl 2-methylacetoacetate, levulinic acid, benzylmalonic acid, 4-hydroxybenzoic acid, p-cresol and benzil.

**Table 1:** The characteristic urinary metabolites selected by OPLS-DA model, SAM analysis and Mann-Whitney U test for discriminating gastric cancer from healthy controls

Metabolites (OPLS-DA)	VIP	SAM*	Mann-Whitney U*
Up-regulated			
Serine	1.91	●	*
Isoleucine	1.90	●	*
Proline	1.88	●	*
Propanedioic acid	1.85		*
Phenylalanine, carbamic acid,	1.80	●	*
Tryptophan	1.72	●	*
Ethyl 2-methylacetoacetate	1.72	●	*
2,3-Octanedione	1.70	●	*
Glycine	1.69	●	*
Levulinic acid	1.63	●	*
4-Hydroxybenzoic acid	1.61	●	*
Valine	1.60	●	*
Tryptamine	1.55		*
Benzil	1.54	●	*
Threonine	1.54	●	*
Tyrosine	1.53	●	*
p-cresol	1.53		*
Butanedioic acid, ethylidene-	1.49		*
Alanine	1.46	●	*
Methionine	1.39	●	*
Benzylmalonic acid	1.32		*
Down-regulated			
5-methyl-hexene	1.43		*
2,2,6,6-tetramethyl-4-oximinopiperdin-1-oxyl	1.39		*
Carbamic acid, ethyl	1.40		*
Hippuric acid	1.33	●	*
3-Pyridinecarboxamide	1.23		*

* The black point means that the corresponding metabolite was chosen by SAM-analysis. The black star means that the corresponding metabolite differs significantly between healthy control and gastric cancer patient by Mann-Whitney U test.

### Validation of 17 selected metabolites in validating set

**Figure 2:**
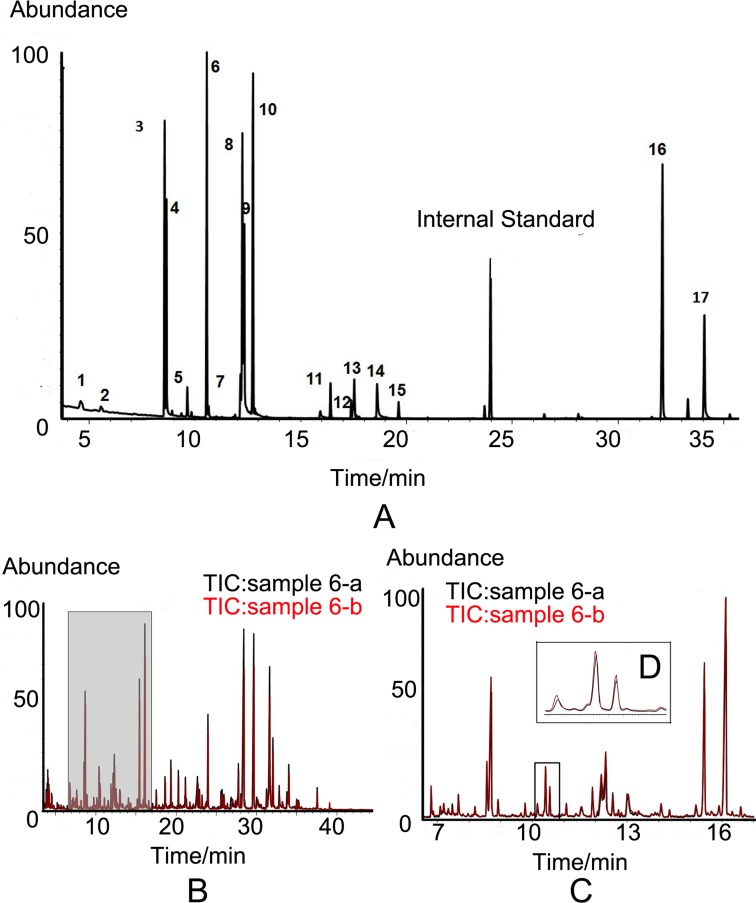
TIC chromatogram **A.** TIC chromatogram of the 17 candidate standards by GC-MS. The peak numbers from 1 to 17 represent ethyl 2-methylacetoacetate, levulinic acid, alanine, glycine, p-cresol, valine, serine, isoleucine, threonine, proline, benzylmalonic acid, 4-hydroxybenzoic acid, methionine, hippuric acid, benzil, tyrosine and tryptophan. L-2-chlorophenylalanine was used as an internal quality standard. **B.** The overlay of two TIC chromatograms from one sample. **C.** Enlargement of TIC from 7 to 17-min. **D.** Further enlargement of regional peaks.

**Figure 3:**
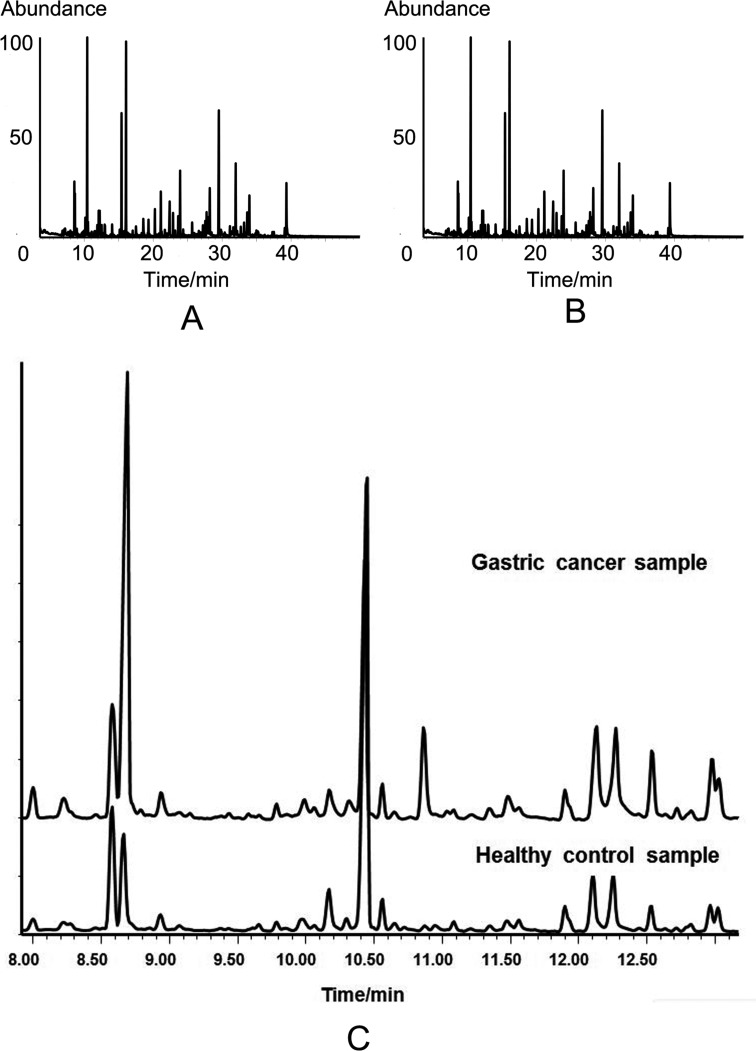
TIC chromatograms of urine samples from different groups by GC-MS analysis **A.** TIC chromatogram of healthy control. **B.** TIC chromatogram of gastric cancer patient. **C.** Enlargement of TIC chromatogram at 7.9-min to 13.1-min for comparison of healthy control with gastric cancer patient.

We quantitatively examined the levels of 17 metabolites in urine samples, including 10 amino acids and 7 organic small molecules. A total of199 urine samples were enrolled in the study. Of them, 112 samples were from gastric cancer (including 37 cases of early gastric cancer), and 87 were healthy controls. The candidate metabolites were validated through external standard method. The chemicals, retention time, linear range of concentration and linear fitting coefficients of 17 standard chemicals are summarized in Table 2. The total ion current (TIC) chromatogram of the standard chemicals was presented in Figure 2A.The TIC chromatograms of urine samples derived from the healthy controls and gastric cancer are obtained by GC-MS analysis. The peaks of 17 compounds were identified by the spectrums of the known standards (Supplementary Figure 1, 2, 3). The amount of metabolites depended on the efficiency of chemical derivatization. We randomly detected twice for one sample by two different operators at interval of 48 hours and found that the two TIC profiles of same sample were overlapped well, which reflected the stability and reliability of GC-MS analysis (Figure 2B, 2C and 2D). We also tested the recovery of standards in urine to validate our methodology to analyze complex compounds. A 300μl standard solution with 5, 10, 20 and 40μg/ml of 17 compounds was spiked to 300μl urine, prior to ECF-derivatization. The extraction recovery was calculated and the mean recovery of 17 compounds with different concentrations ranged from 81.1 to 107.3% with the relative standard derivation (R.S.D) lower than 9% (Table 3). By TIC analysis, gastric cancer disclosed significant difference from healthy control (Figure 3A, 3B). This difference was more distinct at enlarged TIC chromatogram from 7.9 to 13.1min (Figure 3C).

**Table 2:** The chemicals, retention times, linear range of concentration and linear fitting coefficients of 17 standard chemicals

Chemicals	Retention time(min)	Linear range(μg/600μl)	r2*
Amino acids			
L-Alanine	8.582	0.02-40	0.999522
Glycine	8.668	0.02-40	0.999713
L-Valine	10.567	0.02-40	0.999729
L-Serine	12.197	0.50-40	0.997635
L-Isoleucine	12.228	0.02-40	0.999611
L-Threonine	12.343	0.02-20	0.998707
L-Proline	12.742	0.02-40	0.999963
L-Methionine	17.536	0.002-4	0.999849
L-Tyrosine	31.984	0.02-40	0.999713
L-Tryptophan	33.986	0.02-40	0.999409
Organic acids			
Ethyl 2-methylacetoacetate	4.602	0.002-2	0.99963
Levulinic acid	5.515	0.002-4	0.998062
p-cresol	9.652	0.002-4	0.999038
Benzylmalonic acid	16.391	0.002-4	0.999243
4-Hydroxybenzoic acid	17.348	0.002-4	0.999731
Hippuric acid	18.586	0.1-40	0.998476
Benzil	19.580	0.002-2	0.996025

* Regression coefficient

**Table 3:** Recovery of the 17 standards

Chemicals	Recovery (%)*	
	Average	R.S.D
Alanine	92.6	4.0
Glycine	86.9	3.6
Valine	85.6	5.2
Isoleucine	98.6	7.0
Serine	94.5	3.8
Threonine	96.6	4.9
Proline	88.8	4.2
Methionine	83.8	5.3
Tyrosine	81.1	5.0
Tryptophan	85.5	4.5
Ethyl 2-methylacetoacetate	87.3	3.53
Levulinic acid	89.0	4.0
p-cresol	85.9	4.4
Benzylmalonic acid	98.5	8.7
4-Hydroxybenzoic acid	87.7	3.6
Hippuric acid	104.7	7.91
Benzil	107.3	3.8

* Mean recovery was obtained by 16 determinations (four parallel samples at four different concentrations) with an internal standard.

### Diagnostic value analysis of 17 urinary metabolites

**Figure 4:**
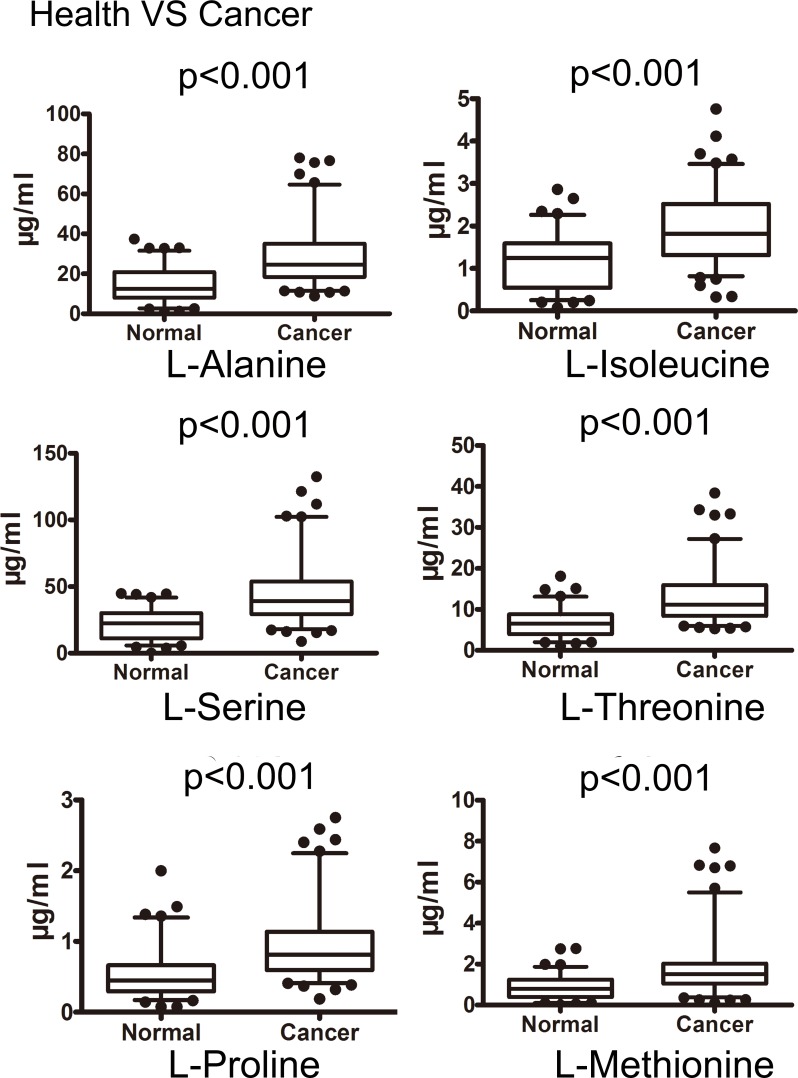
Box plots of the levels of potential urinary biomarkers that could distinguish cancer from controls The p-values of Mann-Whitney U test were indicated (the concentration unit μg/ml).

**Figure 5:**
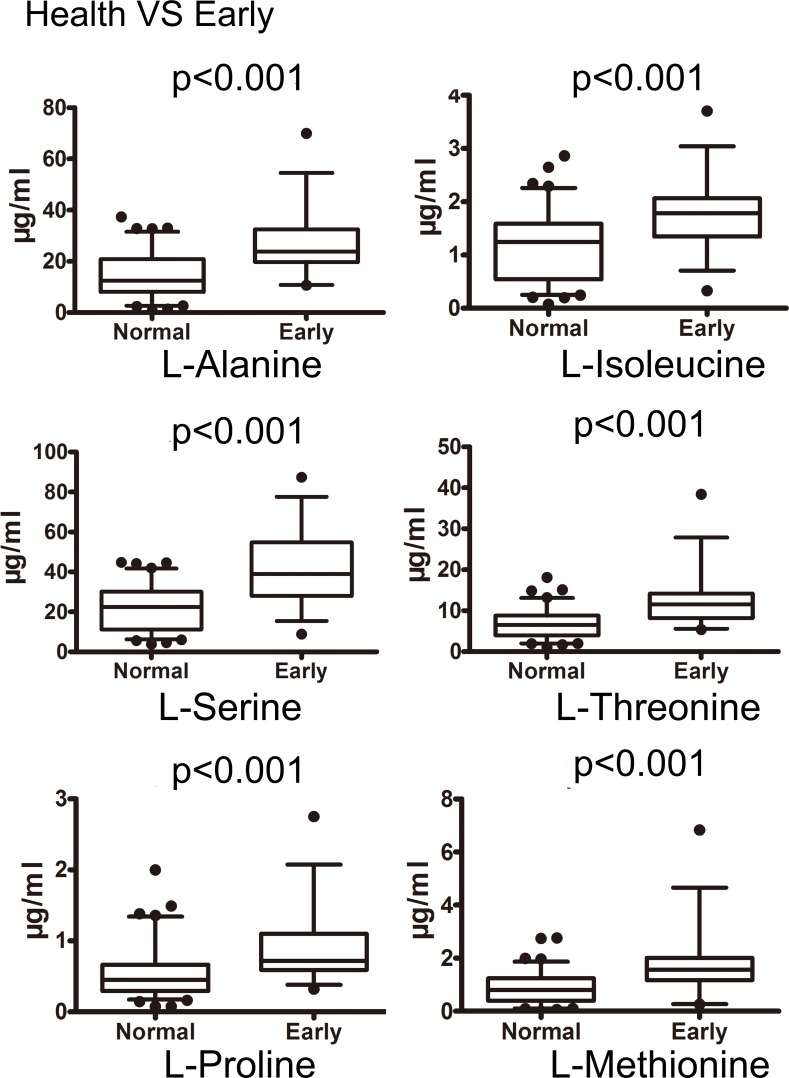
Box plots of the levels of potential urinary biomarkers that could distinguish early gastric cancer from controls The p-values of Mann-Whitney U test were indicated (the concentration unit μg/ml).

**Figure 6:**
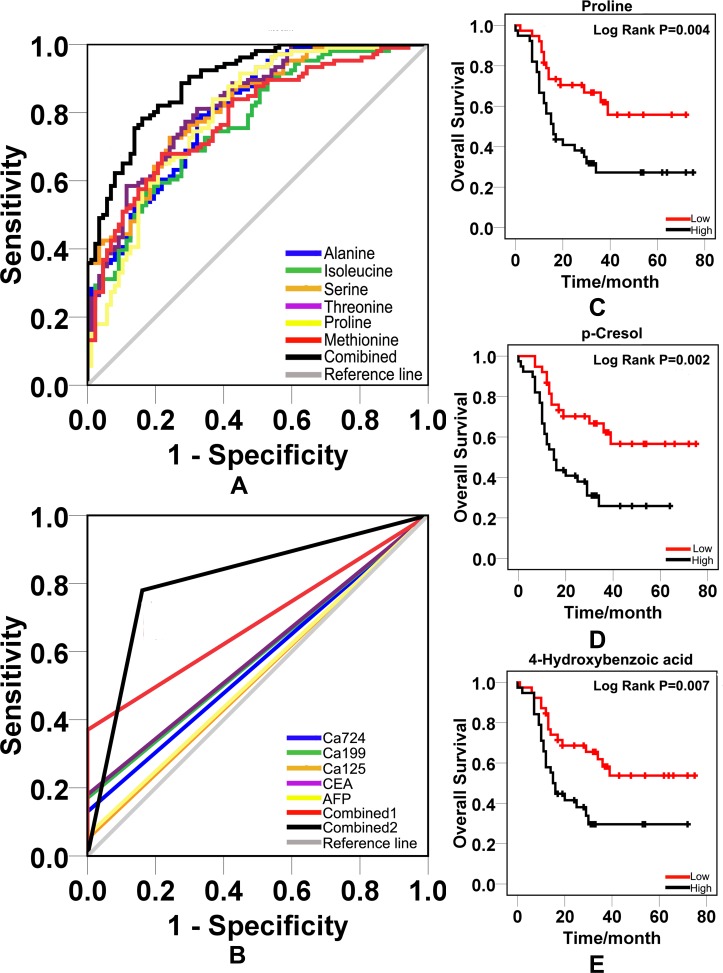
ROC curves and survival curves **A.** ROC curves were obtained from 14 increased urinary metabolites and their combination. **B.** ROC curves were obtained from classic blood biomarkers and their combination. Combined 1 was the combination of classic blood tumor markers. Combined 2 was the combination of 14 new potential urinary biomarkers. **C.** The survival curve of proline. **D.** The survival curve of p-cresol. **E.** The survival curve of 4-hydroxybenzoic acid.

By Mann-Whitney U test, gastric cancer group could be clearly discriminated by candidate metabolites except for 4-hydroxybenzoic acid, benzil and hippuric acid. This difference was also observed between healthy control and early gastric cancer group. Intensive analysis indicated that the concentration of metabolite p-cresol closely correlates with cancer stage. The levels of p-cresol are gradually increased with patients’ stages (Supplementary Figure 4). The median and interquartile range (IQR) of each candidate metabolite in healthy control, gastric cancer group and early gastric cancer group were listed in Table 4. Fourteen out of 17 candidates (82.35%) revealed diagnostic value on ROC analysis. Six of them revealed satisfactory diagnostic values with area under ROC curve (AUC) more than 0.75. The box charts of these 6 urinary candidate metabolites between healthy controls and gastric cancer groups or early gastric cancer samples are plotted (Figure 4, Figure 5). Their ROC curves were shown in Figure 6A.

**Table 4:** The median and interquartile range(IQR) of urinary metabolite levels (μg/ml)

Metabolite	Healthy	Cancer	*P* value	Early cancer	*P* value
Alanine	12.615(11.553)	24.514(16.555)	<0.001	23.794(12.689)	<0.001
Glycine	42.042(42.262)	63.654(50.772)	<0.001	63.490(46.492)	<0.001
Valine	2.770(2.486)	4.090(2.333)	<0.001	4.150(1.760)	<0.001
Isoleucine	1.243(1.047)	1.813(1.207)	<0.001	1.783(0.717)	<0.001
Serine	22.351(18.974)	39.046(24.530)	<0.001	38.926(26.711)	<0.001
Threonine	6.573(4.820)	11.162(7.503)	<0.001	11.556(5.973)	<0.001
Proline	0.450(0.373)	0.810(0.540)	<0.001	0.717(0.510)	<0.001
Methionine	0.797(0.843)	1.500(0.983)	<0.001	1.567(0.837)	<0.001
Tyrosine	7.969(9.202)	12.062(7.153)	<0.001	11.689(5.289)	0.002
Tryptophan	9.599(8.243)	13.489(8.949)	<0.001	13.209(7.586)	0.004
Molecule 1	0.803(0.683)	1.200(0.917)	<0.001	1.167(0.727)	<0.001
Molecule 2	0.450(0.577)	0.747(0.660)	<0.001	0.793(0.590)	0.001
Molecule 3	0.040(0.033)	0.070(0.093)	<0.001	0.053(0.060)	0.047
Molecule 4	0.107(0.113)	0.137(0.160)	<0.001	0.143(0.123)	0.013

Molecules 1 to 4 represent ethyl 2-methylacetoacetate, levulinic acid, p-cresol and benzylmalonic acid, respectively.

The AUC, sensitivity, specificity and Youden value were listed in Table 5. The cutoff value for each metabolite was determined on the basis of the Youden index [J, J = max {sensitivity + specificity-1}] [31]. We created logistic regression model by binary logistic regression analysis. Zero (health control) or one (gastric cancer) serves as dichotomous variable, and fourteen differential metabolites as the covariates. The predicted equation was as follows:

**Table 5:** The sensitivity, specificity, Youden index, cutoff value, and AUC of each metabolite

Metabolite	Sensitivity	Specificity	Youden	Cutoff	AUC
Alanine	0.783	0.678	0.461	18.268	0.804
Glycine	0.915	0.414	0.329	35.603	0.744
Valine	0.623	0.735	0.358	3.586	0.734
Isoleucine	0.670	0.724	0.394	1.517	0.770
Serine	0.726	0.759	0.485	30.227	0.814
Threonine	0.811	0.678	0.489	8.016	0.823
Proline	0.840	0.632	0.472	0.560	0.793
Methionine	0.679	0.782	0.461	1.260	0.784
Tyrosine	0.858	0.472	0.330	7.179	0.693
Tryptophan	0.821	0.517	0.338	9.676	0.698
Molecule 1	0.538	0.804	0.342	1.163	0.715
Molecule 2	0.651	0.644	0.295	0.583	0.673
Molecule 3	0.726	0.621	0.347	0.043	0.698
Molecule 4	0.472	0.839	0.311	0.177	0.669
Combination	0.774	0.851	0.625	0.555	0.893

Molecules 1 to 4 represent ethyl 2-methylacetoacetate, levulinic acid, p-cresol, benzylmalonic acid, respectively.

P = 1/ [1+e-^(0.222ala-0.019gly-1.334val+2.375iso+0.173ser+0.709thr+2.844pro+4.040met-0.793tyr-0.044try+0.693a+0.417b+14.801c-0.157d-3.853)^]. The letters a, b, c and d represent ethyl 2-methylacetoacetate, levulinic acid, p-cresol and benzylmalonic acid, respectively. The P referred to the value of predicted probability of each sample based on the levels of 14 candidate metabolites. We used the values of predicted probability as new variables and produced a combined ROC curve with a cutoff value 0.555. The corresponding sensitivity was 0.774 and specificity was 0.851. The AUC was 0.893(Figure 6A).

We further compared diagnostic values of urinary molecules with classic blood tumor biomarkers CEA, CA19-9, CA72-4, CA12-5 and AFP on validating set. The raising of any blood tumor biomarkers in one sample was considered as positivity. We established a combined ROC curve of blood tumor biomarkers. We created ROC curves of CEA, CA19-9, CA72-4, CA12-5 and AFP, respectively. The AUC of single blood biomarker ranged from 0.525 to 0.590. The combination AUC was 0.685. However, the combined AUC of urinary molecules was 0.810, which is superior to the combination AUC of classic blood biomarkers (Figure 6B).

### The prognostic value analysis of 17 urinary metabolites

We got follow-up information on 82 out of 112 gastric cancer cases in validating set. The follow-up period was 3 to 5 years after surgery. At the end of follow-up, 35 patients (42.7%) were alive and 47 patients (57.3%) died. We calculated the survival rate based on metabolites levels using Kaplan-Meier method. The differences in overall survival between two groups were compared by log-rank tests. We found that high levels of proline, p-cresol and 4-hydroxybenzoic acid revealed worse survival rate (P < 0.01) with median survival time 16-mons, 15-mons and 15-mons respectively (Figure 6C, 6D and 6E). The median survival time was over 60 months when patients revealed lower levels of proline, p-cresol and 4-hydroxybenzoic acid in urine. Other metabolites were not associated with the prognosis. By hazard ratio (HR) analysis, the HR of p-cresol, proline and 4-hydroxybenzoic acid was 2.688 (95% CI: 1.403-5.150), 2.473(95%CI: 1.293-4.728) and 2.335(95% CI: 1.229-4.435), respectively.

### Metabolic pathway analysis

**Figure 7:**
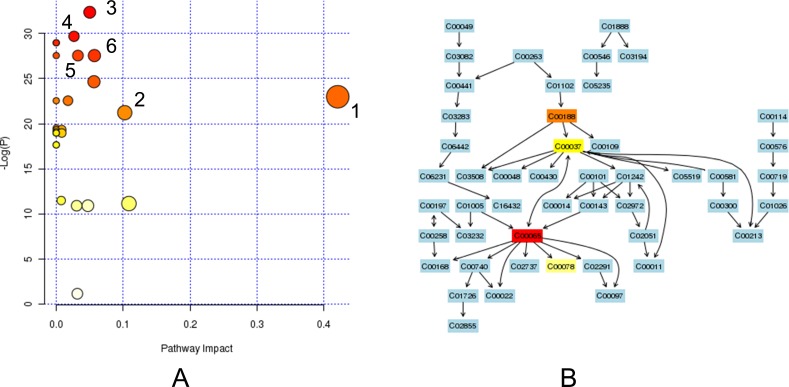
The metabolic pathway analysis **A.** All enrolled pathways by MetPA analysis. The node color is based on P value and the node radius is determined based on their pathway impact values. 1. glycine, serine and threonine metabolism. 2. arginine and proline metabolism. 3. cysteine and methionine metabolism. 4. Valine, leucine and isoleucine biosynthesis. 5.Taurine and hypotaurine metabolism. 6. Alanine, aspartate and glutamate metabolism. **B.** The glycine, serine and threonine metabolism pathway. The map was generated using the reference map by KEGG. CO represents the entry number of chemicals. C00065: Serine, C00188: threonine, C00037: glycine, C00078: tryptophan. The matched chemicals showed different heat map colors based on their P values.

We analyzed the relevant metabolic pathways of gastric cancer by MetPA tool. The identified compounds were distributed in 27 pathways (Supplementary Table 2). The main metabolic pathways included glycine, serine and threonine metabolism (glycine, serine and threonine), arginine and proline metabolism (proline), cysteine and methionine metabolism (methionine, alanine and serine), valine, leucine and isoleucine biosynthesis (threonine, isoleucine and valine), taurine and hypotaurine metabolism (alanine), alanine, aspartate and glutamate metabolism (alanine) (Figure 7A).We noticed that the pathway of glycine, serine and threonine metabolism were excessively activated in gastric cancer (Figure 7B).

## DISCUSSION

Early diagnosis of gastric cancer is crucial to improve patient′ outcome. Once the patient was diagnosed at early stage, he or she will obtain timely treatment [32].Current diagnosis of gastric cancer relies on imaging, endoscopy and histological pathology. Although these examinations are specific and accurate, they are invasive, expensive and therefore not suitable for population screening. Classic blood tumor biomarkers such as CEA, AFP, CA72-4 and CA19-9 are not sensitive enough for gastric cancer diagnosis. Therefore, new diagnostic procedures with better sensitivity and specificity are needed. Here we describe a promising noninvasive procedure. We could obtain the whole metabolites profile on 300μl urine sample within 40 minutes.

Chan and coworkers ever reported urinary metabolomics of gastric cancer [4]. They identified three discriminatory metabolites, 2-hydroxyisobutyrate, 3-indoxylsulfate and alanine. Another study analyzed 154 urine samples from gastric cancer and healthy controls. They found a group of metabolites related to amino acid and altered lipid metabolism. The sensitivity of metabolites is much higher than that from blood biomarker CA19-9 and CEA [28]. However, the sample sizes of previous studies are not large enough, and the diagnostic capability is limited. Up-to-date, none of the urinary metabolomics studies for gastric cancer involved in patient′ prognosis. To our knowledge, this is the largest sample size for urinary metabolomics study on gastric cancer. To characterize the specific metabolites, three rigorous algorithms were used for metabolites selection. We paid attention to metabolites appeared in three algorithms. Finally, 17 metabolites were selected for further validation on validation set. By quantitative detection through constructing standard curves, 14 out of 17 candidates were confirmed. They are alanine, glycine, valine, isoleucine, serine, threonine, proline, methionine, tyrosine, tryptophan, ethyl 2-methylacetoacetate, levulinic acid, benzlmalonic acid and p-cresol. The 14 variables are significantly increased in urine of gastric cancer patients and revealed diagnostic values with AUC from 0.669 to 0.823. The combined AUC of 14 variables reached to 0.893. We further got the cutoff value for each metabolite, which are useful as candidate biomarkers in clinic. Importantly, the 14 candidate metabolites also increased significantly in early gastric cancer patients. Compared to classic blood biomarkers of CEA, AFP, CA72-4, CA19-9 and CA12-5, the urinary metabolites showed better diagnostic values. Particularly, three metabolites could predict the patient′ prognosis. Higher levels of proline, p-cresol and 4-hydroxybenzoic acid were associated with poorer prognosis. Our new findings suggest urinary metabolites could be an alternative for gastric cancer screening.

By metabolic pathways analysis, the pathway of glycine, serine and threonine metabolism were excessively activated in gastric carcinogenesis. Jain and colleagues ever assayed metabolites profiles from media of NCI-60 cancer cell lines and found disorder of glycine consumption, which is correlated with proliferation rate of cancer cells [33]. Hirayama and coworkers [34] reported increased levels of amino acids in colon and gastric cancer tissues. Mayers and coworkers reported elevation of serum branched-chain amino acids in human pancreatic adenocarcinoma [35]. Chan and colleague [4] also found elevated alanine in the urine of gastric cancer patients. Obviously, disturbed amino acids levels in cancer tissue or body fluids are common in gastric cancer. Increased amino acids may due to robust metabolic adaptations for oxidative stress and cell growth.

However, whether the identified metabolites are gastric-cancer-specific or not remains unknown. Some reports disclosed an elevated urinary p-cresol in colorectal cancer [13], valine in bladder cancer [36], proline in ovarian cancer [37], glycine, threonine and tyrosine in liver cancer [18, 38]. Obviously, one single metabolite may not be specific for gastric cancer, but the multiple metabolites combination may increase the specificity for gastric cancer. Therefore, it is necessary to collect various types of cancer for further comparison study.

## MATERIALS AND METHODS

### Collection of urine samples

A 5 ml urine sample from 293 individuals was collected in the early morning before breakfast at Ruijin hospital, School of Medicine, Shanghai Jiao Tong University. Of the 293 subjects, 159 were subjected to primary gastric cancer while the other 134 were controls who visited the hospital for physical examination with age and gender-paired to the patients. Patients with benign digestive diseases such as gastritis, gastric ulcer, or with metabolic diseases such as diabetes, hyperthyroidism were excluded. All of cases enrolled in the research showed normal liver and kidney function by blood biochemistry assay. The malignant diagnosis was confirmed by histopathology after biopsy or operation. The gastric cancer patients with metastases were diagnosed by image examination. Tumors were staged according to the UICC/AJCC TNM Classification (Version 7). None of the patients was on any neoadjuvant chemotherapy before surgical treatment. All samples were obtained under the informed consent. The urine was centrifuged at 3000 rpm for 10 min after obtaining. The supernatant and sediment were aliquoted separately into eppendorf tubes and then stored at -80 OC until use. The samples were divided into two sets. One was for training set with 47 urine samples from healthy controls and 47 urine samples from gastric cancer patients. Another was for validating set with 87 urine samples from healthy controls and 112 urine samples from gastric cancer patients. The age, gender and tumor stage for all patients are provided in Table 6. This study was approved by Hospital Institutional Review Boards for human subject research.

**Table 6:** Baseline characteristics of the samples

Characteristic	Training set			Validating set	
	Healthy		Cancer	Healthy	Cancer
Number	47		47	87	112
Age(median, range)	57/25-80		55/22-78	58/24-83	59/27-87
Male/female ratio	25/22		27/20	46/41	61/51
TNM stage					
StageIa/b		-	13	-	37
StageIIa/b		-	12	-	23
StageIIIa/b/c		-	10	-	24
StageIV		-	12	-	28

### Chemicals

ECF, pyridine, anhydrous ethanol, sodium hydroxide, chloroform, and anhydrous sodium sulfate were analytical grade from China National Pharmaceutical Group Corporation (Shanghai, China). The standard reagents of alanine (Ala), glycine (Gly), isoleucine (Ile), valine (Val), praline (Pro), serine (Ser), threonine (Thr), methionine (Met), tyrosine (Tyr), tryptophan (Try), hippuric acid, ethyl 2-methylacetoacetate, levulinic acid, benzylmalonic acid,4-hydroxybenzoic acid, p-cresol and benzil were purchased (J&K Scientific, USA). L-2-chlorophenylalanine (Shanghai Hengbai Biotech. Co. Ltd., China) was used as an internal quality standard. Ethyl 2-methylacetoacetate, 4-hydroxybenzoic acid, benzil and hippuric acid were diluted in the anhydrous ethanol. Tyrosine was diluted in hydrochloricacid. Others were prepared in the ultra pure water from a Milli-Q system (Millipore, USA).

### Sample derivatization

The process of sample derivatization was conducted as described before [29]. In brief, diluted urine sample (urine: water = 1:1, v/v) or each 600-μl aliquot of standard mixture was added to glass tube. After adding 100 μl of 1-2-chlorophenylalanine (0.1 mg/ml), 400 μl of anhydrous ethanol, and 100 μl of pyridine to the urine sample, 50 μl of ECF was added for first derivatization at 20.0 ± 0.1°C. The mixtures were sonicated at 40 KHz/s for 60 s. Subsequently, exaction was performed using 300 μl of chloroform. The derivatization procedure was repeated with the addition of 50-μl ECF into the aforementioned products. After the two success derivatization, the overall mixtures were vortexed for 30 s and centrifuged for 3 min at 3000 rpm. The aqueous layer was aspirated off, while the remaining chloroform layer containing derivatives were isolated and dried with anhydrous sodium sulfate and subsequently subjected to GC-MS analysis.

### GC-MS analysis

The extracts of derivatization were analyzed with a7890A gas chromatograph coupled with a5975C mass spectrometer (Agilent technologies Inc, USA). A 1-μl extract aliquot of the extracts was injected into a DB-5MS capillary column coated with 5% diphenyl cross-linked 95% dimethylpolysiloxane (30 m×250 μm, 0.25-μm film thickness; Agilent J&W Scientific, Folsom, CA) in the split mode (3:1). Either the injection temperature or the interface temperature was set to 260°C; and the ion source temperature was adjusted to 200°C. Initial GC oven temperature was 80°C. Two-min after injection, the GC oven temperature was raised to 140°C with 10°C/min, to 240°C at rate of 4°C/min, to 280°C with 10°C/min again, and finally held at 280°C for 3 min. Helium was the carrier gas with a flow rate set at 1 ml/min. The detection was conducted with electron impact ionization (70 eV) in both the full scan mode and selective ion monitoring (SIM) scan mode (30-550 m/z). We set the ion as follows: l-alanine 44m/z, l-glycine 102m/z, l-valine 144m/z, l-serine 132m/z, l-isoleucine 158 m/z, l-threonine 101m/z, l-proline 142m/z, l-methionine 61m/z, l-tyrosine 107m/z, l-tryptophan 130m/z, ethyl 2-methylacetoacetate 74m/z, levulinic acid 99m/z, cresols 107m/z, benzylmalonic acid 131m/z, 4-hydroxybenzoic acid 121m/z, benzil 105 m/z, hippuric acid 134m/zandl-2-chlorophenylalanine 102m/z.

### Data analysis

TICs and fragmentation patterns were acquired using GC/MS ChemStation Software (Agilent Technologies, Palo Alto, CA, USA). In training set, the compound identification was performed by comparing the mass spectrum with a standard mass spectrum in the national institute of standards and technology mass spectra library (NIST) with a similarity of more than 70% and verified by available standard compounds. The relative peak area of each compound would be calculated as the level of corresponding compound. For the holistic treatment of these data, multivariate analysis was used to identify the metabolomics differences between the groups. The OPLS-DA was carried out with SIMCA-p software (v 12.0; Umetrics), while SAM analysis was performed with MEV software (http://statweb.stanford.edu/∼tibs/SAM/index.html). Regarding to OPLS-DA, the VIP is considered to be differentiating variables. VIP scores indicate the relative importance of each metabolite in a given OPLS-DA model. Metabolites with a VIP > 1.0 are more influential and thus contribute more to discriminating disease groups [39]. By SAM analysis, the false discovery rate (FDR) was set at 0.01 and the permutation was set at 1000, while the delta value was set at 1.5. By Mann-Whitney U test, the metabolites with P < 0.01 were selected as candidate biomarkers. We integrated the outputs from the three numerations and chose 17 metabolites as candidate biomarkers for subsequent validation.

In validating set, the levels of 17 candidate compounds were calculated by external standard method through constructing standard curves. The sample information, peak intensities and peak retention time are applied for pattern recognition. The concentration of 17 candidate compounds was expressed by median, interquartile range and box chart. The Mann-Whitney U test was used for variables analysis between different groups. The classical tumor markers of CEA, CA19-9, CA72-4, CA12-5 and AFP were examined by chemiluminescence immunoassay (CLIA, Abbott ARCHITECT i2000) or electrochemiluminescence immunoassay (ECLIA, Roche Ecl 2010) during routine blood examination. The ROC curve, AUC, binary logistic regression and Kaplan-Meier analysis were performed with SPSS software (v20, IBM, USA). Metabolic pathway and function analysis was performed by MetPA's tool, which is a web-based metabolomics tool for pathway analysis (http://metpa.metabolomics.ca/MetPA/faces/Home.jsp).

## SUPPLEMENTARY MATERIALS FIGURES AND TABLES




