# Supplementary material for: A characteristic biosignature for discrimination of gastric cancer from healthy population by high throughput GC-MS analysis
Source: Oncotarget. 2016 Aug 31;7(52):87496–510. doi: 10.18632/oncotarget.11754 (PMC5350005; doi:10.18632/oncotarget.11754)
Supplement: Supplementary file 1 [file oncotarget-07-87496-s001.pdf]

A characteristic biosignature for discrimination of gastric cancer from healthy population by high throughput GC-MS analysis

Supplementary Material

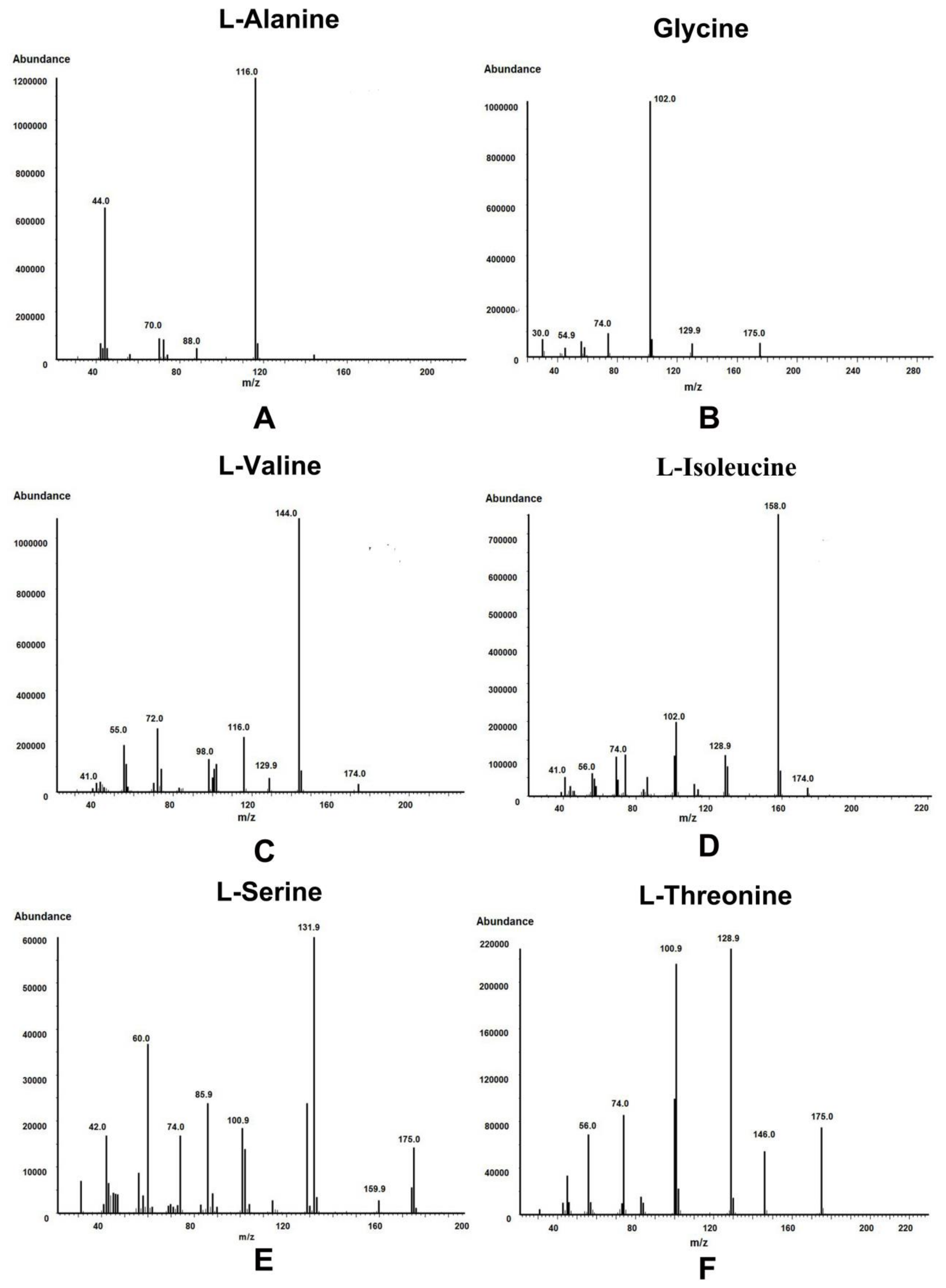

Fig S1. The peaks of compounds alanine, glycine, valine, isoleucine，serine and threonine were identified by the spectrums of the known standards.

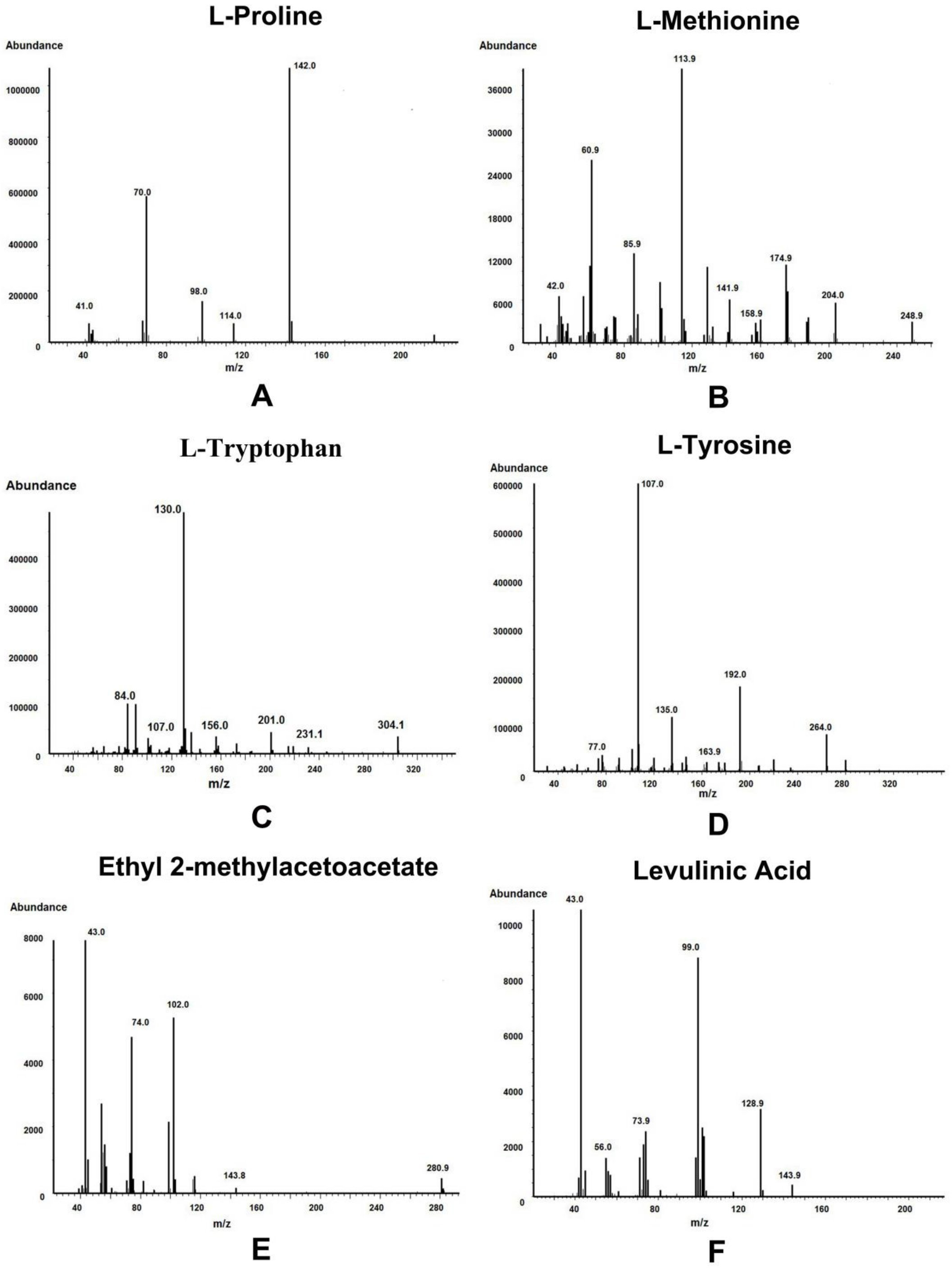

Fig. S2. The peaks of compounds praline, methionine, tryptophan, tyrosine, ethyl 2-methylacetoacetate and levulinic acid were identified by the spectrums of the known standards.

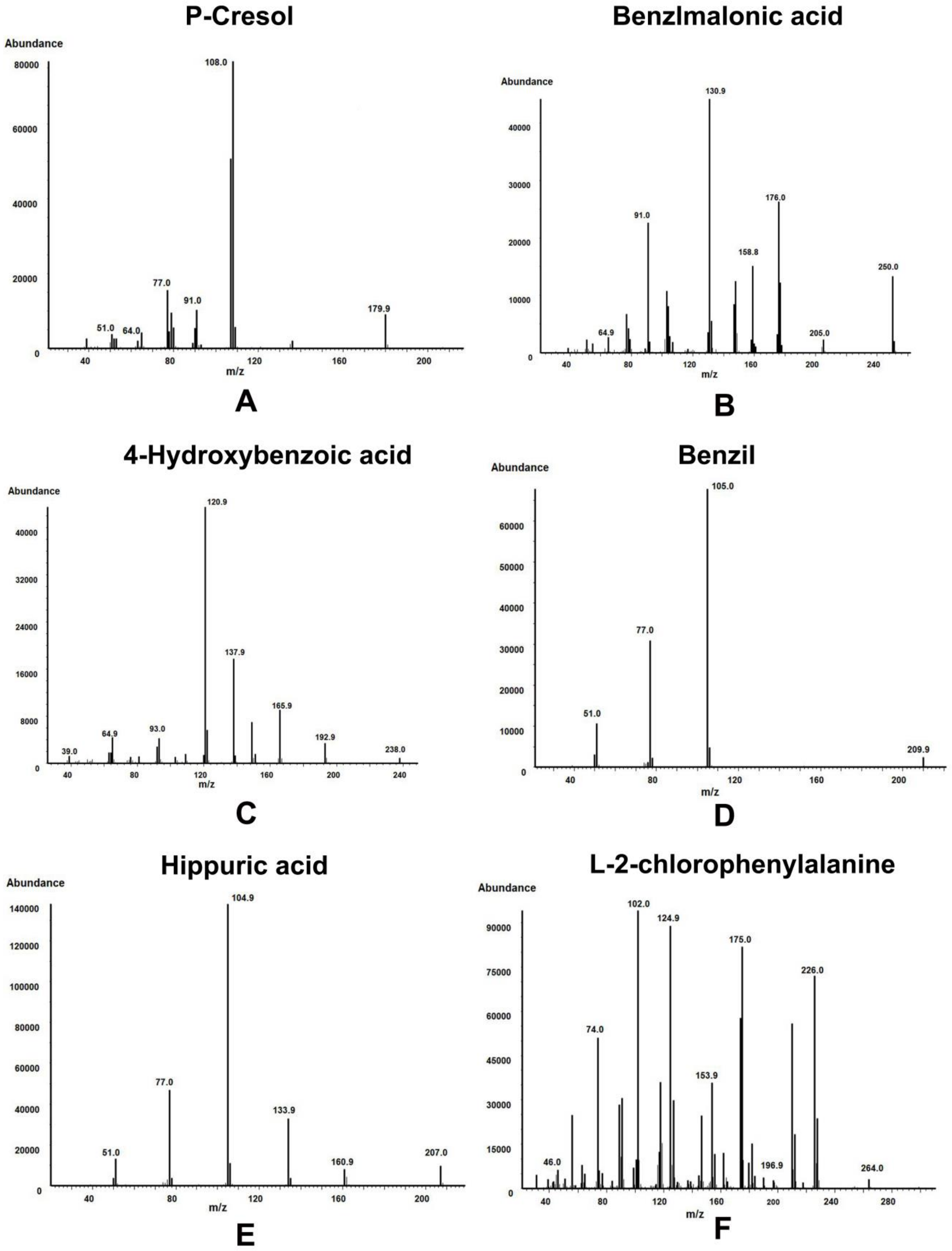

Fig. S3. The peaks of compounds p-cresol, benzlmalonic acid, 4-hydroxybenzoic acid, benzil, hippuric acid and L-2-chlorophenylalanine were identified by the spectrums of the known standards.

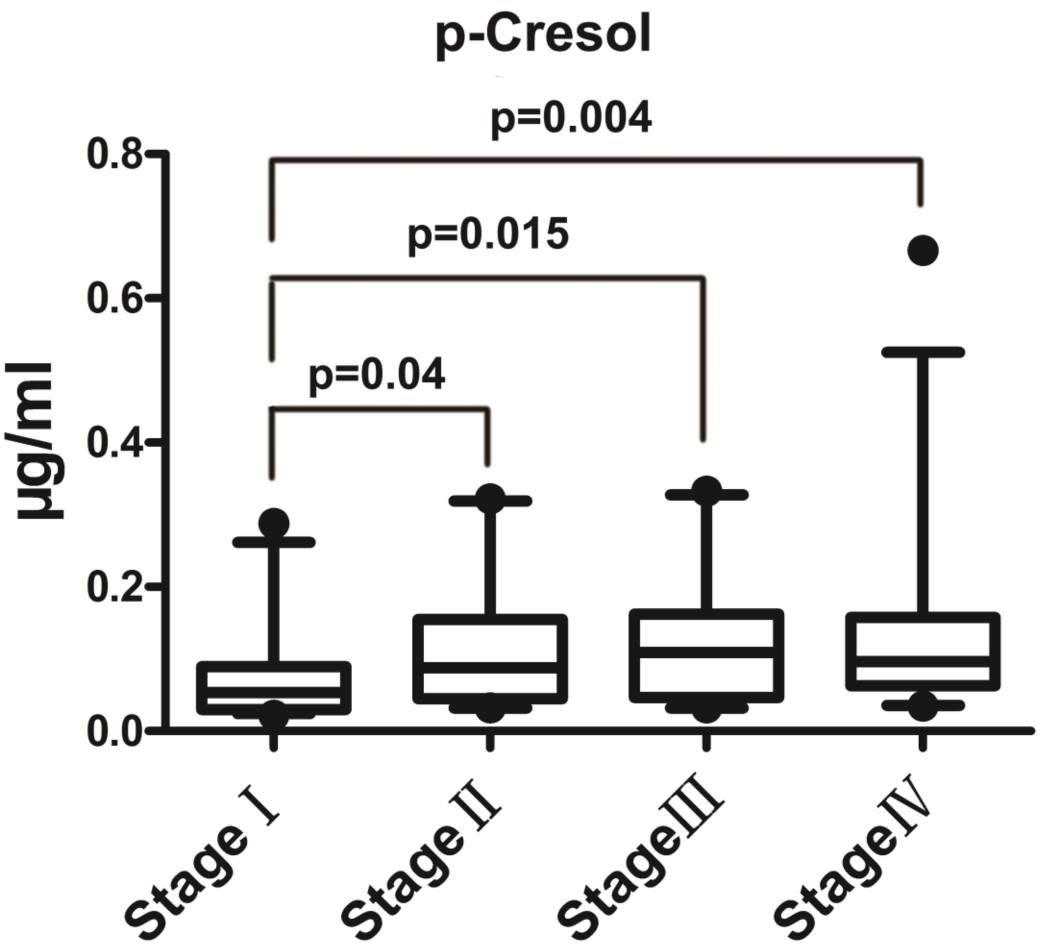

Fig. S4. The relationship of p-cresol level with patient’s stages. The levels of p-cresol are gradually increased with patients’ stages.
