# Supplementary material for: A characteristic biosignature for discrimination of gastric cancer from healthy population by high throughput GC-MS analysis
Source: Oncotarget. 2016 Aug 31;7(52):87496–510. doi: 10.18632/oncotarget.11754 (PMC5350005; doi:10.18632/oncotarget.11754)
Supplement: Supplementary file 2 [file oncotarget-07-87496-s002.docx]

**Supplemental Table 1. Small compounds identified in urine of gastric cancer**

| NO | RT(min) | Compounds |
| --- | --- | --- |
| 1 | 3.653 | Carbamic acid, dimethyl |
| 2 | 3.755 | Carbamic acid, ethyl- |
| 3 | 3.848 | Butanoic acid, 3-hydroxy- |
| 4 | 3.979 | Acetoacetic Acid |
| 5 | 4.053 | 3-Hydroxy-3-methylbutanoate |
| 6 | 4.174 | Urethane |
| 7 | 4.202 | O-ethyl-l-homoserine |
| 8 | 4.221 | Butanoic acid, 2-methyl- |
| 9 | 4.230 | Benzaldehyde |
| 10 | 4.572 | Phenol |
| 11 | 4.602 | Butanoicacid,2-methyl-3-oxo- |
| 12 | 4.680 | 2,3-Octanedione |
| 13 | 5.422 | Butanoic acid, 2-(hydroxymethyl)- |
| 14 | 5.515 | Pentanoic acid,4-oxo- |
| 15 | 5.589 | propanedioic acid |
| 16 | 5.720 | p-Cresol |
| 17 | 6.008 | Propanedioic acid,methyl |
| 18 | 6.083 | Tetrahydro-6-methyl-2h-pyran-2-one |
| 19 | 6.464 | Hexanoic acid,3-hydroxy |
| 20 | 6.800 | 2,2,6,6-Tetramethyl-4-oximinopiperdin-1-oxyl |
| 21 | 6.865 | 4-Octenoic acid |
| 22 | 7.089 | Propanedioic acid, ethyl- |
| 23 | 7.238 | Butanedioic acid |
| 24 | 7.463 | 4-Pyridinecarboxylic acid |
| 25 | 7.575 | Butanedioic acid, methyl- |
| 26 | 7.800 | 1-Piperidinecarboxylic acid |
| 27 | 7.996 | Itaconic acid |
| 28 | 8.174 | Benzeneacetic acid |
| 29 | 8.233 | Succinoic acid, 2-hydroxy-3-methyl |
| 30 | 8.492 | Malic acid |
| 31 | 8.576 | Alanine |
| 32 | 8.660 | Glycine |
| 33 | 9.007 | Indole |
| 34 | 9.437 | Glutaconic acid |
| 35 | 9.549 | 3-Methylglutaconic acid |
| 36 | 9.652 | p-Cresol |
| 37 | 9.783 | Alanine, N-methyl- |
| 38 | 9.951 | n-(1-Methyl ethyl)-l-valine, methyl |
| 39 | 10.045 | 2,4-Dimethyl-3-hepanone |
| 40 | 10.363 | Hexanedioic acid |
| 41 | 10.56 | Valine |
| 42 | 10.831 | Benzenebutanoic acid, γ-oxo-, |
| 43 | 10.85 | Acetamide,n-(3-methyl-2-buten-1-yl)- |
| 44 | 10.953 | 5-Propionyloxy-pent-3-enoic acid |
| 45 | 11.074 | Heptanedioic acid |
| 46 | 11.091 | 4,4-Dimethyl-3-oxo-pentaneoic acid |
| 47 | 11.346 | l-Valine, N-(carboxymethyl)- |
| 48 | 11.411 | Butanedioic acid, ethylidene-, |
| 49 | 11.467 | 2-Pyrrolidinecarboxylic acid-5-oxo- |
| 50 | 11.561 | 2-(Ethoxycarbonyloxy) carbamate |
| 51 | 11.897 | Leucine |
| 52 | 11.977 | 2-t-Butyl-5-ethylidene-6-methyl-[1,3]dioxan-4-one |
| 53 | 12.052 | Pentanedioic acid |
| 54 | 12.197 | Serine |
| 55 | 12.225 | Isoleucine |
| 56 | 12.343 | Threonine |
| 57 | 12.372 | Heptanedioic acid |
| 58 | 12.44 | 2,15-Hexadecanedione |
| 59 | 12.524 | Citric acid |
| 60 | 12.711 | Proline |
| 61 | 13.010 | Benzonitrile, 2-nitro- |
| 62 | 13.038 | 2-Hydroxy-2,3-dimethylsuccinic acid |
| 63 | 13.263 | Cyclohexanecarboxylic acid |
| 64 | 13.824 | dl-α-Methylglutamic acid |
| 65 | 14.048 | Aspartic acid |
| 66 | 14.158 | Hexanedioic acid |
| 67 | 14.329 | Suberic acid |
| 68 | 14.463 | Dibutyl phthalate |
| 69 | 14.494 | Dodecanoic acid |
| 70 | 15.143 | Heptanedioic acid, 4-oxo-, |
| 71 | 15.442 | Propene-1,2,3-tricarboxylic acid |
| 72 | 15.658 | 3-Pyridinecarboxylic acid,ethyl ester |
| 73 | 15.845 | Butanoic acid, 2-methyl- |
| 74 | 16.032 | Citric acid |
| 75 | 16.295 | 1-Aminocyclopentanecarboxylic acid |
| 76 | 16.391 | Benzlmalonic acid |
| 77 | 16.592 | Cyclohexanebutanoic acid |
| 78 | 17.002 | 2-Nitro-2-cyclohexene-1-acetic acid |
| 79 | 17.025 | Glutamic acid |
| 80 | 17.096 | Ethylparaben |
| 81 | 17.352 | Ethylparaben |
| 82 | 17.536 | Methionine |
| 83 | 17.682 | Dibutyl phthalate |
| 84 | 18.264 | 1-Oxaspiro[4.5]dec-3-en-6-ol, 6,10,10-trimethyl-, acetate |
| 85 | 18.358 | n-Methyl-3-pyridinecarboxamide |
| 86 | 18.586 | Hippuric acid |
| 87 | 18.738 | Benzeneacetic acid, 3-hydroxy- |
| 88 | 19.347 | Benzeneacetic acid, 4-hydroxy- |
| 89 | 19.58 | Benzil |
| 90 | 19.853 | Carbonic acid, ethyl 4-isopropylph |
| 91 | 20.264 | Phenylalanine |
| 92 | 20.733 | 1,2-Benzenedicarboxylic acid, bis(2-methylpropyl) ester |
| 93 | 20.895 | 2,3,6-Trideoxy-3-[(trifluoroacetyl)amino]hexopyranose |
| 94 | 21.073 | n-(Phenylacetyl)-glycine |
| 95 | 21.145 | 1h-Indole-3-acetic acid5-hydroxy- |
| 96 | 21.243 | 1H-Indole-3-acetic acid |
| 97 | 21.265 | 2-Amino-n-(4-nitrophenyl)-3-phenylpropanamide |
| 98 | 21.702 | O-AcetylCitrate |
| 99 | 22.097 | 2,3-Diazabicyclo[2.2.1]heptane-2,3-dicarboxylic acid, 5-methyl-, |
| 100 | 22.204 | Benzamide, 4-ethoxy- |
| 101 | 22.382 | Clemastine |
| 102 | 22.434 | 5-Aminovaleric acid |
| 103 | 22.705 | Heptanedioic acid, 4-oxo- |
| 104 | 23.138 | Butyl phthalate |
| 105 | 23.214 | Homovanillic acid |
| 106 | 23.613 | Hexadecanoic acid |
| 107 | 23.734 | 3,3'-Diaminobenzidine |
| 108 | 23.936 | 2-Chloro-phenylalanine |
| 109 | 26.12 | Benzeneacetic acid, phenyl ester |
| 110 | 26.409 | Glycyl-l-proline |
| 111 | 27.462 | Oleic Acid |
| 112 | 27.717 | Hexadecanamide |
| 113 | 28.287 | l-Aminocyclopentanecarboxylic acid |
| 114 | 28.473 | Benzeneacetic acid, α,4-dihydroxy- |
| 115 | 28.722 | 4-Aminomethyl-pyridine |
| 116 | 29.672 | Valine, N-(2,4-dinitrophenyl)- |
| 117 | 30.079 | Glutamine, N2-(phenylacetyl)- |
| 118 | 31.26 | 4-Ethoxybenzhydryazide |
| 119 | 31.321 | n-(2-hydroxybenzoyl)-glycine |
| 120 | 31.521 | Tyrosine, n-carboxyl,cyanomethyl,ester |
| 121 | 31.643 | 5-Hydroxyindole-3-acetaldehyde |
| 122 | 31.88 | Tryptamine |
| 123 | 31.984 | Tyrosine |
| 124 | 32.324 | Benzoic acid, 2,4-diethoxy |
| 125 | 32.696 | Prolylglycine |
| 126 | 33.685 | Indol-3-acetic acid,5-hydroxy |
| 127 | 33.986 | Tryphton |
| 128 | 35.364 | unknown |
| 129 | 39.085 | Cysteine |

Supplementary Table 2. Result from ingenuity pathway analysis with MetPA

| **No.** | **Pathway Name** | **Total*** | **Hits**** | **Impact***** |
| --- | --- | --- | --- | --- |
| 1 | Cysteine and methionine metabolism | 56 | 3 | 0.05 |
| 2 | Valine, leucine and isoleucine biosynthe­sis | 27 | 3 | 0.03 |
| 3 | Sphingolipid metabolism | 25 | 1 | 0.00 |
| 4 | Sulfur metabolism | 18 | 1 | 0.00 |
| 5 | Alanine, aspartate and glutamate metabolism | 24 | 1 | 0.06 |
| 6 | Taurine and hypotaurine metabolism | 20 | 1 | 0.03 |
| 7 | Selenoamino acid metabolism | 22 | 1 | 0.00 |
| 8 | Aminoacyl-tRNA biosynthesis | 75 | 10 | 0.06 |
| 9 | Glycine, serine and threonine metabolism | 48 | 4 | 0.42 |
| 10 | Methane metabolism | 34 | 2 | 0.02 |
| 11 | Cyanoamino acid metabolism | 16 | 2 | 0.00 |
| 12 | Arginine and proline metabolism | 77 | 1 | 0.10 |
| 13 | Valine, leucine and isoleucine degrada­tion | 40 | 2 | 0.00 |
| 14 | Porphyrin and chlorophyll metabolism | 104 | 2 | 0.00 |
| 15 | Nitrogen metabolism | 39 | 3 | 0.00 |
| 16 | Thiamine metabolism | 24 | 2 | 0.00 |
| 17 | Primary bile acid biosynthesis | 47 | 1 | 0.01 |
| 18 | Purine metabolism | 92 | 1 | 0.00 |
| 19 | Lysine degradation | 47 | 1 | 0.00 |
| 20 | Glutathione metabolism | 38 | 1 | 0.00 |
| 21 | Propanoate metabolism | 35 | 1 | 0.00 |
| 22 | Pantothenate and CoA biosynthesis | 27 | 1 | 0.00 |
| 23 | Phenylalanine, tyrosine and tryptophan biosynthesis | 27 | 2 | 0.01 |
| 24 | Tryptophan metabolism | 79 | 1 | 0.11 |
| 25 | Ubiquinone and other terpenoid-quinone biosynthesis | 36 | 2 | 0.03 |
| 26 | Tyrosine metabolism | 76 | 1 | 0.05 |
| 27 | Phenylalanine metabolism | 45 | 3 | 0.03 |

*Total means the number of compounds in the pathway. **Hits is the actually matched number from the user uploaded data. ***Impact is the pathway impact value calculated from pathway topology analysis.
